# From young aces to injury cases: a qualitative study of stakeholder perspectives on injury risk in junior to elite badminton

**DOI:** 10.1186/s40798-026-01107-9

**Published:** 2026-10-11

**Authors:** Brid Stepper, Lars Donath, Hendrik Stagge, Oliver Faude, Anne Hecksteden

**Affiliations:** 1 Institute of Sport Science, University Innsbruck, Innsbruck, Austria; 2 Department of Training Intervention Research, German Sport University Cologne, Cologne, Germany; 3 Department of Sport, Exercise and Health, University of Basel, Basel, Switzerland; 4 Institute of Physiology, Medical University Innsbruck, Innsbruck, Austria; 5 Department of Applied Exercise Science, German Sport University Cologne, Cologne, Germany

**Keywords:** Injury risk, Badminton, Racket sports, Qualitative sports research

## Abstract

**Background:**

Competitive badminton is associated with a considerable burden of injuries, particularly during the transition from junior to elite level (ages 16–20). While the sport's dynamic stroke and movement patterns may contribute to injury risk, the factors stakeholders use to assess injury vulnerability remain poorly understood. However, identifying specific causes and indicators that point to an increased risk of injury is essential for prevention. Given the limited case numbers, combining data driven approaches with stakeholders’ experiential knowledge may enhance risk assessment. This study explores expert judgement and heuristics regarding injury risk assessment as well as options for risk mitigation during the transitional phase.

**Methods:**

Twenty semi-structured in-person interviews were conducted with national-level stakeholders (9 coaches, 5 athletes, 2 association doctors, 2 physiotherapists, 2 strength and conditioning coaches) between October 2023 and July 2024. The interviews had an average duration of 54.4 min. Thematic analysis helped identify common patterns across assessments.

**Results:**

Four higher-order themes were identified: Excessive pressure, recovery deficits, unprofessional attitude, and disruptions in training continuity. Six cue families were perceived by stakeholders as informing injury risk assessment: signs of over-motivation, unprofessional attitude, the coaches’ subjective perception (‘coaches’ eye’), upheavals in private life, exposure and signs of wear. While overlaps with the literature were found, stakeholders often downplayed commonly cited objective factors like injury history. Many cues were based on intuitive, experience-driven judgments and were therefore difficult to standardize or quantify.

**Conclusions:**

Experts relied on relative risk judgments shaped by experience and situational impressions rather than absolute probabilities. Integrating these practitioner-derived qualitative cues with objective data may enhance context-sensitive injury risk assessment during the junior-to-elite transition. By making experiential heuristics more transparent, the findings offer a foundation for developing more targeted injury mitigation strategies in this critical developmental phase. Further empirical validation is required.

**Supplementary Information:**

The online version contains supplementary material available at https://doi.org/10.1186/s40798-026-01107-9.

## Key points

- Injury risk is high in competitive badminton, especially during the transition from junior to elite level (16–20 years), a period that stakeholders associate with increased injury vulnerability.
- Interviewees described several experiential cues, including over-motivation, deviations from habitual movements or behaviour and upheavals in private life, which they considered important when assessing injury vulnerability.
- The study highlights the crucial role of the coach's subjective perception in the early injury detection and prevention.

**Supplementary Information:**

The online version contains supplementary material available at https://doi.org/10.1186/s40798-026-01107-9.

## Introduction

With a shuttlecock speed of up to 565 km/h [1], badminton is the fastest racket sport in the world [2]. Badminton is characterised by numerous actions in which high intensity actions alternate with longer phases of lower intensity during the rally breaks [3–5]. During the rallies, there are many accelerations, decelerations and changes of direction. Complex interactions between technical, tactical, physiological and psychological skills are crucial [6].

Although badminton is not a contact sport, both acute and overuse injuries are common due to typical repetitive movement patterns [7]. Numerous lunges, jumps, quick changes of direction and overhead strokes put a lot of strain on both the lower and upper extremities [7]. The most commonly reported injuries involve the lower extremities, particularly the ankles and knees, followed by the shoulder, back and trunk [8]. The injury incidence ranges between 1 and 4 injuries/1000 h [8], with some studies reporting injury incidences of up to 7 injuries/1000 h [9]. The range of recorded injury rates is wide, even within the top-level player segment. Overall, the reported incidence rates place badminton within the range observed in other competitive sports (for comparison, football, 13–19-year olds: 1–5 injuries/1000 h training [10]). However, the injury profile in badminton is characterised by a comparatively high proportion of overuse injuries, reflecting the cumulative mechanical demands of repetitive movement patterns [8].

Studies have identified the transition age between junior (between the ages of 16 and 20, in badminton training at a youth training centre) and elite level (over 20 years old and training at an adult national training centre) as being particularly prone to injury [9, 11, 12]—regardless of the type of sport. In badminton, the authors justify the peak in this age group by saying that the mechanical strain of playing increases with increasing skill, which in turn ‘grows’ with players age [13]. Also at this age, there are many risk factors in both the private and sporting environment. For example, many players move away from home to train within the national high-performance system and transition from youth training centres to senior elite environments, which is often accompanied by a sudden increase in training volume and intensity as well as new organisational and social demands. At the same time, young athletes must balance sporting development with educational transitions and increasing performance expectations. Taken the negative consequences of injuries for the health and performance of badminton players into account, it seems imperative to understand, assess and minimise the risk of injury.

Contemporary injury research conceptualises injury development as a complex and context-dependent process. Frameworks such as the ‘web of determinants’ model [14], Finch’s TRIPP framework [15], and more recent contextual extensions of the van Mechelen model [16] emphasise that injury risk emerges from dynamic interactions between biological, psychological, and environmental factors, among others. This perspective highlights that understanding injury vulnerability requires attention not only to isolated risk factors but also to the broader socioecological context in which athletes are embedded.

Sports injury research has traditionally been guided by quantitative epidemiological frameworks that aim to understand the causes and mechanisms of injury (‘inference’), estimate individual injury risk based on available data (‘prediction’), and evaluate and implement evidence-based preventive measures such as targeted training, recovery strategies, or protective equipment (‘mitigation’) [17]. These approaches have substantially advanced knowledge about injury epidemiology and prevention and are reflected in widely used frameworks such as the van Mechelen model [18], which outlines a four-step approach to sports injury prevention. However, they do not fully capture the experiential, contextual, and interpretive processes through which practitioners assess injury vulnerability in everyday practice. The present study therefore complements existing epidemiological approaches by exploring how experienced stakeholders perceive, interpret, and integrate different sources of information when judging injury risk.

Sports injury research faces several methodological challenges: limited case numbers, numerous interacting and influencing factors and a probabilistic injury outcome in which chance plays a role [19]. These factors limit the predictive power of scientific studies [20] and thus also their immediate practical applicability. To find new solutions, it is crucial to recognise that quantitative studies are not the only source of knowledge. Empirical knowledge and human judgement also play an important role in practical decision-making [21, 22]. In sport particularly, the combination of scientific knowledge and the experiential knowledge of experts—including implicit knowledge and intuitive heuristics—can create valuable synergies [20, 23].

While established epidemiological models provide an important basis for injury surveillance and prevention, they rely primarily on quantifiable indicators. Qualitative and quantitative approaches address complementary aspects of injury research. While these frameworks have substantially advanced knowledge of injury epidemiology, they do not fully capture qualitative dimensions such as stakeholders' perceptions, experiences, and belief systems that may shape how injury risk is recognised and managed in everyday practice [22, 23]. The present study takes a qualitative approach to examine experiential and contextual knowledge [16] that may not be fully captured by traditional epidemiological measures. This methodology has already been applied in football by Hecksteden et al. (2026) [24].

Previous injury research has increasingly recognised the importance of contextual and organisational influences on injury prevention [25–28]. However, these studies have primarily used quantitative or mixed-methods designs and therefore address different research questions than the present study. Rather than identifying epidemiological associations, our qualitative approach aims to understand how experienced stakeholders perceive, interpret, and integrate diverse sources of information when assessing injury risk.

**Fig. 1:**
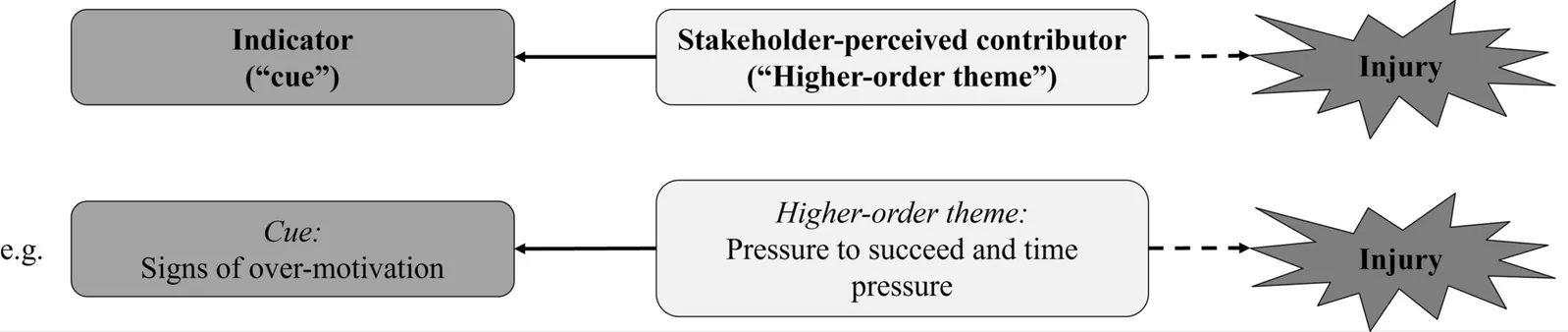
Top: Abstract visualization of a theme map showing a stakeholder-perceived contributing theme for a potential injury, which is often latent, together with its observable indicator (“cue”). Bottom: Exemplary illustration of the relationship between higher-order themes and cues, showing the effect of pressure. The relationships shown are based on stakeholder perceptions and are intended as a conceptual representation rather than a formal causal pathway analysis

Based on these considerations, we conducted expert interviews with stakeholders in competitive youth badminton. We aimed to gain insights into experiential knowledge and expert judgement concerning the factors that contribute to the risk of injury during the transition from junior to adult professional badminton. We further distinguished broader themes describing perceived contributors to injury vulnerability from observable cues (manifest or latent) that stakeholders use during injury risk assessment (see Fig. 1). Semi-structured interviews and subsequent thematic analysis were used to identify cues for an increased risk of injury in adolescent badminton players. The focus was on understanding the cues and heuristics of the experts, which can make an important contribution to improving risk assessment. In complex environments where data is limited or incomplete, practitioners often rely on heuristics—that is, simplified decision-making strategies or ‘shortcuts’—to make quick decisions based on experience and contextual clues [23, 29].

In this context, it is essential to clearly distinguish between higher-order themes and cues (see Fig. 1). Themes refer to broader, higher-order concepts that stakeholders associated with increased injury vulnerability. They are typically used in retrospective or inferential analysis to understand why an injury may have occurred. In contrast, cues are specific, often situationally observed or intuitively perceived indicators that experts use in the moment to make judgements about an individual's injury risk. Cues do not necessarily explain the injury risk, but they serve as practical heuristics in real-world decision-making, especially in dynamic and time-constrained environments. By investigating which cues are used in practice and how they relate to broader themes, this study aims to gain insights into the knowledge and judgement processes that underpin expert assessments of injury risk. This knowledge may contribute to more nuanced, context-sensitive approaches to injury prediction and prevention in elite youth sport.

## Methods

### General approach

The study was approved by the local ethics committee (Ethics Committee German Sport University Cologne, Ethics Application No. 189/2023) and complies with the Declaration of Helsinki [30]. Written informed consent was obtained from all participants prior to the interviews. We confirm that the team of authors, and in particular the interviewers, HS and BS, have no collaboration (previous or ongoing), dependency or personal relation (beyond acquaintance) with the interviewees. The key part of this study consists of semi-structured interviews with stakeholders of competitive youth badminton (expert elicitation). The aim of the study was to explore how stakeholders perceive and interpret cues and contextual factors that may signal increased injury vulnerability during the transition from junior to elite badminton. It is important to note that the higher-order themes identified in this study represent stakeholders' subjective perceptions of factors that may contribute to injury risk. They should therefore be understood as conceptual categories derived from participants' experiences rather than objectively established mechanisms of injury. This study is grounded in an interpretivist qualitative paradigm, assuming that injury risk assessments are shaped by context-dependent experiences and subjective meaning-making. Thematic analysis using the Braun and Clarke's six-phase model [31] was used to identify and interpret areas of overlap (explicit and latent) between the experts' views. To contextualise the insights gained from the interviews, relevant scientific literature, particularly in the field of badminton epidemiology and associated indicators, was consulted [32–49].

In the interviews, we asked participants to share their personal experiences regarding important indicators for assessing injury risk in junior-to-elite transition athletes in badminton. While the coaches provided an external perspective based on their observations of the athletes, the players themselves reflected on their own experiences with injury. An injury was defined as a health problem that interrupts normal training for the athlete concerned and causes a loss of time [50, 51].

### Interviews

#### Interviewees

People from professions within the coaching staff (badminton coaches in junior competitive sport, strength and conditioning coaches specialized in badminton, association doctors and physiotherapists) were interviewed. Multiple stakeholder groups were included to capture complementary perspectives on injury risk assessment within the transition phase. These persons had to have worked with athletes of transitional age at the highest German level for at least 5 years. We also interviewed (former) players who had played or were still playing badminton as a competitive sport for at least 15 years and were playing or had played at least at the highest national level. We recruited interviewees from each of the officially accredited youth training centres in Germany (in seven different locations), as well as athletes from elite performance centres. Anyone who met our criteria and wanted to participate was allowed to do so. Finally, we recruited 20 interview partners from the German Badminton Association who meet these high criteria. The interviews took place between October 2023 and July 2024.

#### Interview framework

A semi-structured interview was applied. There were predefined questions as a guideline (see Appendix A), which had to be addressed. However, there was room for following the interviewees line of thought before coming back to the questions prescribed by the guideline. At the beginning, we examined our basic assumption in the interviews that the transitional years between junior and elite sport level, i.e., athletes aged 16–20, are particularly at elevated risk of injury.

The interviewees were then asked to imagine the following competition: The person (e.g., within a coaching team) who can best predict an impending injury within the next 28 days in players from their training group on the basis of cues wins (hypothetical ‘prediction tournament’). The players were asked to answer the questions drawing on all sources of information available to them. This included their own experiences with injury, observations of injuries within their training group, as well as any additional knowledge or impressions they may have gained.

The interviews explored perceived injury risk in the transition phase (U17–U22), the feasibility of individual risk prediction, and the most relevant cues used in practice. To support cue prioritisation, participants sorted keyword cards (see Appendix B) on a visual analogue scale according to perceived importance after answering the open-ended questions. A photo of this was taken at the end. In this study, the scale was only analysed qualitatively. Further questions addressed contextual factors influencing cue relevance, personal experiences with injury, current practices at the training centre, and potential areas for improvement. Finally, they were asked to briefly report on their personal background and their connection to badminton. The wording of the questions was slightly adapted depending on the role of the interviewees (players, doctors/ physiotherapists, coaches) so that the players could report from their internal perspective and the coaches from an external perspective.

The interviews were conducted exclusively in person face-to-face. The interviews were recorded using a dictation recorder, with a target duration of 60 min.

#### Thematic analysis

Thematic analysis was conducted to identify and interpret overlaps in the experts' statements [31]. This type of data evaluation is a principled yet flexible and pragmatic analytical approach that makes it possible to identify patterns of meaning from a qualitative data set (e.g., respondents' answers from an interview). Both explicitly and implicitly mentioned themes and subthemes were taken into account. Care was taken to maintain an inductive perspective throughout and focus on the interview data, while limiting interpretation in the light of the epidemiological paradigm.

We followed the six-phase model of thematic analysis described by Braun and Clarke (2006) [31]. Authors BS and HS familiarised themselves with all interview transcripts. The coding was carried out using MAXQDA 24 software (VERBI Software, 2024). BS and HS first coded the interview transcripts separately in MAXQDA, then merged them and developed a preliminary set of themes and cues, which were discussed and revised by the entire author team until consensus was reached. In a final step, the themes and cues were given concise titles and definitions by AH and BS.

## Results

### Interviewees

A total of 20 interviews were conducted and analysed. 15 people (12 male, 3 female) came from the coaching staff, including 2 association doctors, 2 physiotherapists, 2 strength and conditioning coaches and 9 badminton coaches who work at youth training centres. The remaining 5 people (3 male, 2 female) are both former and current top-level players at least at the highest German level. The interviews had an average length of 54.4 min (range: 29.6–78.5 min).

### Interview results

#### Injury risk and the transition age to elite badminton

The respondents agreed on their experiences, confirming our assumption that the risk of injury is higher during the transition age. They justify this by pointing out that both the intensity and volume of training increase significantly at this age, but also that their own playing level rises, leading to more intense training sessions and competitions. The interviewees describe ‘adult badminton’ as a ‘different sport’ compared to lower age groups due to the significantly higher intensity. This effect is often intensified by the age-related change from the junior to the national centres after leaving school. There, the volume and intensity of training is increased in a short time frame, meaning that there is hardly any chance of familiarisation with the new training environment. The young players also have to get used not only to the physical demands, but also to the changed living conditions after leaving school. Therefore, according to some interviewees, it is the responsibility of the coaches to adequately prepare the players for the change.

#### Higher-order themes for an increased risk of injury

Table 1 summarises the interview results in the form of higher-order themes and subthemes representing stakeholder-perceived contributors to injury vulnerability in junior competitive badminton. Direct quotations can be found in the supplements (Appendix C). The frequency table is provided for transparency regarding the distribution of higher-order themes across interviews and can be found in Appendix E.

**Table 1:** Overview of the higher-order themes of coaching staff and players—stakeholder-perceived contributors

**Higher-order theme 1: Excessive pressure**
There was a consensus that excessive pressure massively increases an athlete's risk of injury, whether caused by the athletes own expectations, externally by parents or coaches or by time constraints
a) Sub-theme 1.1: Intrinsic pressure to perform Own expectations create intrinsic pressure. As a result, athletes may train more than is actually sensible (attempt to “try with the crowbar”)
b) Sub-theme 1.2: Make it or not—external pressure in the transition age External pressure to perform increases, especially in the transition to senior competitive sport. In the early U22 age group, parents are often cited as a factor, but later it is primarily the coaches and the association that pass on the pressure to succeed ‘from above’ to the players
**Higher-order theme 2: Recovery deficits**
a) Sub-theme 2.1: Accumulating load Recovery deficits, triggered by physical exhaustion due to too many competitions in a row or intensive weeks of training, can lead to a high risk of injury. Especially at transition age, players are expected to play many tournaments to show themselves to the national coaches and to achieve the highest possible ranking
b) Sub-theme 2.2: Boom-and-Bust Cycle If the training volume or intensity is increased too quickly, this often exceeds the athlete's exercise tolerance and results in recovery deficits. This can lead to minor (and even major) injuries, which in turn prevent training continuity. After recovery, the athletes often try to catch up again quickly (boom), which again leads to a load spike—and thus to the next injury (bust)
**Higher-order theme 3: Unprofessional attitude**
a) Sub-theme 3.1: Enthusiasm for athletics training is key The predictive value of good athleticism was proactively emphasised by most respondents. As a result, athletic training is just as important as on-court training and should not be neglected by athletes due to an unprofessional attitude. Two aspects were particularly emphasised: The factors ‘strength’ and ‘mobility’ should not be considered separately but must be in a balanced relationship and individual “eternal construction sites” should be trained separately in athletics training
b) Sub-theme 3.2: Lacking athletic mindset Many coaches refer to this as ‘not being an athlete’. The affected athletes often neglect everything that is associated with an athlete's life. Skipping athletic training and an unhealthy lifestyle are the most frequently mentioned
**Higher-order theme 4: Challenges to the continuity of the training process**
a) Sub-theme 4.1: Life events Major changes in the private life can lead to an increased risk of injury. In particular, the demanding transition from school to further education (university, etc.) was frequently mentioned. However, moving from a youth training centre to a national training centre also represents a major change in both sporting and personal terms
b) Sub-theme 4.2: Interruptions in the training process If there are frequent interruptions and breaks in the training process due to illness or minor injuries (‘when it warms up, it’s not as bad’), the risk of an ‘official’ injury increases

There was a consensus that excessive pressure significantly increases the risk of injury for athletes (higher-order theme 1: Excessive pressure). This pressure can arise from their own expectations, external influences such as parents or coaches, or time pressure. Personal expectations lead to intrinsic pressure, which can cause athletes to train beyond what is reasonable (sub-theme 1.1: Intrinsic pressure to perform). The attempt to “try with the crowbar” impedes on a trusting collaboration with the coaches regarding load management as well as honest communication of well-being and minor complaints. This issue was deemed to be particularly relevant after periods of time-loss, e.g., due to injury, school duty or other factors (sub-theme 4.2: Interruptions in the training process). A frequently mentioned situation in which the players' own pressure was a driving force was the theme of “time pressure”. If training is ‘caught up’ after a break (due to holiday/injury/illness)—e.g., because of the ‘deadline’ of an upcoming tournament—the risk of injury increases considerably, according to members of the coaching staff. On the one hand, the athletes try to get back to their physiological level before the break as quickly as possible on their own initiative, but it seems particularly important to them to regain the feeling for the court and the racket at short notice.

The external pressure to perform is also increasing, especially at transitional age (sub-theme 1.2: Make it or not—external pressure in the transition age). Coaches and associations in particular pass on the pressure to succeed from above to the players. For the players, it seems even more important to prove themselves and not show any weakness to the coach to establish themselves in professional badminton. This in turn reinforces the internal pressure.

A lack of recovery, caused by physical exhaustion from consecutive competitions or intense training weeks, increases the risk of injury (higher-order theme 2: Recovery deficits). This is especially critical during the transition age, when players are expected to compete in numerous tournaments to gain visibility with national coaches and improve their ranking (sub-theme 2.1: Accumulating load).

It was often mentioned that the ‘seriousness’ is increasing: Badminton is developing from a ‘hobby’ in the children's sector to a ‘job’ in the adult sector. A rapid increase in the volume or intensity of training can also exceed an athlete's capacity and lead to recovery deficits (sub-theme 2.2: Boom-and-Bust Cycle). This often occurs when changing to a new training environment. As a result, minor or even major injuries can occur that disrupt training consistency. After recovery, athletes often try to catch up on the lost time too quickly (boom), which leads to another peak load and thus increases the risk of another injury (bust). This point is closely linked to sub-theme 1.1 (Intrinsic pressure to perform, “try with the crowbar”) and sub-theme 4.2 (Interruptions in the training process).

Most interviewees emphasised the importance of good athleticism for being injury-free (sub-theme 3.1: Enthusiasm for athletics training is key). Two key aspects stood out: a balance between ‘strength’ and ‘mobility’ and the need to individualise athletics training. If athletes do not do athletics training or only do it irregularly, this was attributed to an unprofessional attitude and ‘not being a real athlete’ (sub-theme 3.2: Lacking athletic mindset).

Significant changes in personal life can elevate the risk of injury (sub-theme 4.2: Interruptions in the training process). The challenging transition from school to higher education (university, etc.) was often highlighted. Additionally, the shift from a youth training centre to a national training centre also represents a major change, both athletically and personally.

Frequent disruptions in the training process caused by illness or minor injuries can increase the likelihood of a more serious injury occurring. This is closely related to sub-themes 1.1 (Intrinsic pressure to perform) and 2.2 (Boom-and-Bust Cycle), especially when players do not allow sufficient time to progressively increase their training load and physical conditioning appropriately but try to get fit at the last minute.

#### Risk indicators and assessment

Table 2 summarises the cues that, in the experience of the interviewees, indicate an increased risk of injury or which the participants perceived as informing their assessment of injury risk. These can be both visible and latent in the sense of not being directly observable/ measurable or only accessible through experience and the so-called coaches' eye. Direct quotations can be found in the supplements (Appendix D). The frequency table is provided for transparency regarding the distribution of cue families across interviews and can be found in Appendix F.

**Table 2:** Cues indicating an increased risk of injury (both visible and latent cues)

**Cue-family 1: Signs of over-motivation**
a) Cue 1.1: Players do additional training (“Trying with the crowbar”) Many interviewees report that players go far beyond the actually planned training and train additional sessions of their own. This is mainly due to internal and external pressure to succeed and time pressure (higher-order theme 1)
b) Cue 1.2: Lack of honest communication It is often mentioned that the self-assessment of one's own well-being is not honestly communicated to the coach. The reasons given for this are usually their own ambition and the associated fear of being taken out of training by the coach (higher-order theme 1), but also the fear of ‘showing weakness’ to the coach
**Cue-family 2: Unprofessional attitude (‘not being an athlete’)**
a) Cue 2.1: Visibly deficient athleticism Coaches often cite the visible basic constitution of an athlete as an important indicator. They often mention the difference between athletes with good muscular definition and those with a more ‘flabby’ physique, which makes them more susceptible to injury
b) Cue 2.2: Unhealthy lifestyle The coaching staff cite the quantity of sleep in particular as a major problem and emphasise that athletes are much more susceptible to injury when they are tired
**Cue-family 3: “Something is different”**
Many coaches state that they can see when something is different, i.e., when there is a deviation from the individual habitual norm. This concerns particularly the aspects below:
a) Cue 3.1: Visible (rapid) changes to the body Significant changes to the body, whether due to a change in diet or a very rapid increase in strength, but also due to natural growth, can indicate an increased risk
b) Cue 3.2: Deviation from habitual movement quality Coaches describe deviations from each athlete's own movement profile, which is known after working with the players for a long time. They often describe ‘out-of-round’ movements that are atypical for the player during the warm-up on the court (“coaches’ eye”)
c) Cue 3.3: Psycho-social deviations from the ‘individual normal state’ Coaches describe all deviations from a player's normal, usual behaviour as conspicuous. They emphasise that you need to know the player well for this. But there can also be deviations from the athlete's habitual interaction style off the court, which the coaches describe as conspicuous, where ‘alarm bells go off’. Some describe it as ‘noticing something in the athlete's eyes’ (‘I can see if something is wrong’) or being able to ‘read something between the lines’ during the conversation
**Cue-family 4: Upheavals in the private life**
Changes in the private life (moving to a new training location, transition from school to university, etc.) can increase the risk of injury. Coaches report that they can find this out in a personal conversation either directly through the athlete's words or through a subjective impression
**Cue-family 5: Exposure**
a) Cue 5.1: New stuff—The danger lies in the unknown This primarily relates to the training content (qualitative). If certain topics are worked on that are new and unfamiliar with this special focus, the susceptibility to injury can increase
b) Cue 5.2: Load spikes If there are major changes in the training plan, e.g., a rapid increase in the load in athletic training, this leads to an increased risk of injury. A rapid increase in training volume as well as in intensity due to a change of training location can make an athlete more susceptible to injury
**Cue-family 6: Signs of wear**
This refers to (minor) overuse symptoms, which are often referred to as ‘signs of wear’ (“when it warms up, it’s not as bad”)

Internal pressure (higher-order theme 1: Excessive pressure) is described by stakeholders as a key underlying factor contributing to increased injury risk during the transition phase. This internal drive can lead to behaviours such as over-motivation (cue-family 1: Signs of over-motivation). If there is time pressure in the form of an upcoming tournament in the near future, this can be intensified. On the one hand, players do additional training in the belief that they can improve their skills shortly before the tournament, but also to ‘not lose’ their feeling for the court and racket (cue 1.1: Players do additional training (“Trying with the crowbar”)).

It is frequently noted that athletes do not honestly communicate their self-assessment of well-being to their coach (cue 1.2: Lack of honest communication). The reasons for this are often driven by personal ambition and the fear of being removed from training (higher-order theme 1: Excessive pressure), as well as the concern of appearing weak to the coach. However, this is merely a latent cue that can only be made visible through closer enquiry and a feeling on the part of the coach.

Stakeholders often view an athlete's visible physical condition as a key indicator (sub-theme 3.1: Visible (rapid) changes to the body). They frequently highlight the distinction between athletes with well-defined muscles and those with a ‘softer physique’, suggesting that the latter may be more prone to injury (cue 2.1: Visibly deficient athleticism). Reasons for a lack of athleticism are seen more in an unprofessional attitude (only wanting to play badminton without doing athletic training) than in other reasons, such as lack of time.

One cue for the unprofessional attitude (cue-family 2: Unprofessional attitude (‘not being an athlete’)) is the unhealthy lifestyle (cue 2.2: Unhealthy lifestyle). Too little sleep was mentioned particularly frequently here. However, this is difficult to detect from the outside and can only be recorded either by a monitoring tool (sub-theme 1.2 “Make it or not—external pressure in the transition age” is of particular importance here) or by the subjective impression of the coach (cue-family 3: “Something is different”). An unhealthy diet is often cited as the second component. However, this cannot be monitored around the clock by the coaching staff. Coaches often rely on their experience and intuition to recognise when something is wrong with an athlete. This can literally happen through the coaches’ eye if there are significant and visible changes in an athlete's physical condition (cue 3.1: Visible (rapid) changes to the body) or if the player is already on the court and the coach judges their movements to be out of round and atypical for the player (cue 3.2: Deviation from habitual movement quality). However, all deviations from habitual behaviour on a psycho-social basis also appear to be cues for an increased risk of injury (cue 3.3: Psycho-social deviations from the ‘individual normal state’). This can become apparent both in personal conversations and when observing behaviour on the court during training. An example of this was described as follows: If a player gets loudly excited in training or competition, even though he is usually the quieter type, this can indicate over-motivation and internal pressure; if a rather loud type of player is quiet on a day, he does not seem to be 100% focussed, which may indicate recovery deficits (higher-order theme 2: Recovery deficits).

Changes in personal life, such as moving to a new training facility or moving from school to university, can increase the risk of injury (cue-family 4: Upheavals in the private life). This can have various consequences, such as losing focus (lack of concentration) or poor recovery (e.g., lack of sleep).

The risk of injury can increase when athletes are exposed to unfamiliar training content or experience significant changes in their training plan (cue-family 5: Exposure). This heightened risk often arises when new training aspects (e.g., focus on jumps) or unfamiliar exercises (e.g., jump rope) are introduced. If these movements or methods are unfamiliar to the athlete, it can lead to greater susceptibility to injury (cue 5.1: New stuff—The danger lies in the unknown). Similarly, a sudden increase in training volume, intensity, or changes in the training environment, such as relocating to a new facility, can result in overload, further elevating the risk of injury (cue 5.2: Load spikes).

Many cite mild overuse injuries (cue-family 6: Signs of wear) as a common problem in competitive sports, but also specifically in badminton with its characteristic jumps, sudden stops, changes of direction, and overhead shots. This can lead to minor complaints that are not yet classified as injuries and are therefore ignored and prolonged, but can later develop into more serious injuries.

One point that many coaches raise, in addition to all the cues, is the diffuseness of many injuries that occur. They emphasise an unclear connection between the risk of injury and the supposedly visible cause and describe this as players who ‘seem to have a lot of risk factors’ but who do not get injured and, conversely, players who at first glance have hardly any visible risk factors but who then get injured and no cause can be suspected afterwards. Some coaches describe an injury in an unforeseeable context as ‘bad luck’ and at the same time emphasise that injuries are part of the risk of playing sport at a competitive level and reaching one's own performance limit.

#### Synthesis of the results

**Fig. 2:**
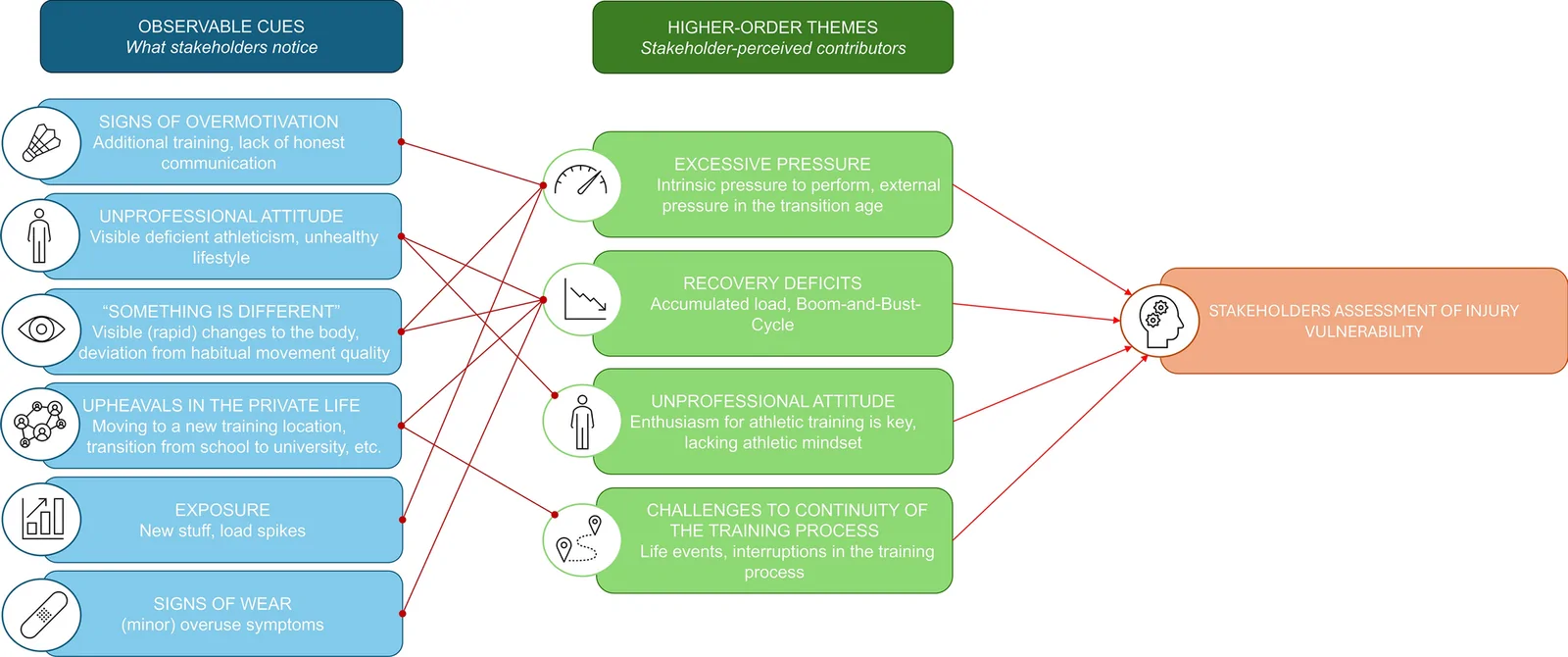
Conceptual summary of the stakeholder-perceived relationships between observable cues, higher-order themes and the assessment of injury vulnerability

The relationships between the identified cue families and higher-order themes are summarised conceptually in Fig. 2. Rather than representing isolated indicators, stakeholders described integrating multiple observable cues into broader higher-order themes, which together informed their overall assessment of an athlete's injury vulnerability.

#### Differences between stakeholder categories

The following was a striking observation made by the interviewers during the interviews: Differences emerged between the various stakeholders with regard to the frequency and distribution of certain higher-order themes and cues being addressed. Physiotherapists and physicians predominantly reported from a retrospective perspective, as they are usually only in irregular contact with athletes—mainly when a health problem has already occurred. Accordingly, their assessments often focused on identifying possible causes after an injury, based on clinical observations and conversations with the athletes.

In contrast, the badminton coaches surveyed, who work closely with the athletes in daily training, showed a more intensive insight into temporal developments and subtle behavioural changes. Accordingly, cues of a psychological or interpersonal nature—such as signs of over-motivation (cue-family 1), deviations from typical behaviour (cue-family 3), or private upheavals (cue-family 4)—were mentioned primarily by the coaches.

The strength and conditioning coaches focused primarily on athletic factors (cue 2.1: Visibly deficient athleticism) as well as training content and sudden peaks in load (cue-family 5: Exposure), which is in line with their role. The athletes themselves largely confirmed the coaches' assumptions regarding dishonest communication (cue 1.2: Lack of honest communication) and attributed this—together with the tendency to complete additional training sessions on their own initiative (cue 1.1: Players do additional training (“Trying with the crowbar”))—to high intrinsic and extrinsic performance pressure (higher-order theme 1).

In addition, another significant difference emerged between individuals with a scientific background and those with a purely practical sports orientation. Several factors that are often highlighted in scientific literature as predictors of injury were rated as less important or not mentioned at all by stakeholders without a scientific background. For example, stakeholders without formal scientific background tended to attach less importance to biomechanical markers (e.g., leg axis) or injury history. Instead, they emphasised interpersonal indicators such as changes in communication patterns, reduced engagement in training sessions or subtle behavioural changes.

## Discussion

This work reveals new insights into how experienced stakeholder in competitive youth badminton assess the risk of injury during the transition from junior to elite sport. The structured elicitation of expert knowledge serves as a complementary source of information to epidemiological research and offers a basis for the development and improvement of formalised risk assessment models.

### Contextualising interview findings with relevant literature

There are numerous studies in the literature on the preventive correlation between athletics training and injuries [32, 33], which is why a visible lack of athleticism (cue 2.1)—a factor that many of our interviewees mentioned as predictive for injury—can conversely be conducive to injury. This is independent of the underlying cause—be it that athletics training cannot be carried out due to a lack of time or due to the aforementioned unprofessional attitude (cue-family 2), which the interviewees cited as a more plausible cause. An unhealthy lifestyle (cue 2.2), combined with a lack of sleep in particular, is also a notable risk factor for injuries in the literature [34]. There is also agreement with cues for an increased risk of injury in the literature with regard to cue-family 5 (Exposure). Large and rapid changes in training load can increase the risk of injury (cue 5.2: Load spikes) [34, 35]. Hulin et al. [35] showed that the risk of injury in rugby players is associated with an increased training load in the week before the injury compared to the average training load of the previous four weeks. In this study, the highest risk of injury was associated with the greatest increase in training load. Major changes in private life (cue 4.1: Upheavals in the private life) combined with a high stress level can also be seen as a predictor variable for injuries [36–38]. Rapid changes to the body (cue 3.1: Visible (rapid) changes to the body), caused by a change in diet, for example, have also been categorised in the literature as causing injury [34].

On the other hand, there are cues that play an important role in the scientific literature but were not rated equally high by the experts. Above all, the short-term (last 28 days) and long-term (entire sports career) injury histories were given very different importance [39, 40]. In general, only a few interviewees mentioned this aspect proactively and only a few people had experience with this point or had the impression that athletes who had already been injured would also suffer from more injuries in the future when the interviewers mentioned the possible importance of pre-injuries. However, if an injury has not been fully cured or has not been adequately followed up, the coaches and players agree that this can be an indicator of a follow-up problem with recurring injuries. The different weighting of the injury history factor between the various professions should be emphasised here. In particular, the on-court coaches, who by their own admission are not very active in sports science or the medical sector, paid less attention to injury history than people from the (medical) support staff with a specific scientific background. The athletes themselves also rarely mentioned the injury history proactively. On the one hand, it is conceivable that in practice there is often a lack of systematic documentation and detailed long-term observation, including medical diagnoses, so that the coaches can see the training and performance, but have little understanding of the long-term relation between previous injuries and new problems. On the other hand, athletes who successfully return after an injury seem to be more present in the coaches' perception than players who drop out or end their career in the long term due to recurring injuries.

In contrast to what is described in the literature [41–43], where the ‘gender’ factor plays a role, the interviewees rated the topic of ‘gender’ as less relevant and as not clearly going in one direction. Many of our interviewees do not recognise any differences between male and female players in terms of the quantity or type of injury. Other few recognise a difference in the type of injured structure. These interviewees say that men are more likely to have muscular injuries, while women are more likely to have ligament and joint injuries. Overall, however, the empirical values differ and there is no clear tendency towards one gender that could be named.

### Important cues are only accessible to the ‘coaches’ eye’

Some very plausible factors that are considered to be important by many stakeholders for the risk of injury (cue 3: “Something is different”) are complex and cannot be assessed directly. This mainly concerns deviations from an athlete's normal state or behaviour, both on a physical level (movement quality) and on a human/social behavioural level. There was a consensus that these factors—at least to date—are only accessible to consciously applied human perception and judgement (‘coaches’ eye’). Many coaches find it difficult to verbalise it and put into words the criteria they include in the coaches’ eye.

Experts therefore rely more often on highly individualised circumstances and an intuitive assessment than on general, measurable cues, such as injury history or quantifiable, objective training load data. In the literature, the coaches’ eye is particularly well known in talent scouting [44–46], where the holistic nature of the coaches' assessment reflects the potential of coaches to integrate information from several different dimensions and assess players more as a whole. This offers clear advantages over other assessment methods (such as motor tests) and leads to easier and at the same time more equal decision-making in terms of the overall assessment when selecting a player [44].

### Integrative perception of latent risk factors—a skill of experts

If an injury is viewed as the result of complex systems [14, 47, 48], the identified higher-order themes and observable cues (from Tables 1 and 2) should be understood as interacting components rather than independent contributors considered in isolation. In most cases, the higher-order themes (Table 1) are latent and therefore not directly measurable or observable in daily training. However, what can become visible through expert knowledge and a trained eye are the indicators (“cue”, Table 2). In our interviews, experts identified external and intrinsic pressure (higher-order theme 1) as a broader underlying theme that was perceived to contribute to increased injury vulnerability but is only latent. Signs of over-motivation (cue-family 1), such as independently organized additional training (cue 1.1), can serve as observable cues that stakeholders interpret as reflecting this higher-order theme.

It seems that the experts often do not explicitly assess the absolute probability of injury (e.g., between 0 and 1). Instead, the risk is assessed relative to a familiar or expected state, which acts like a baseline. This is an implicit comparative value, i.e., a subjective baseline against which deviations are recognized and evaluated. So, if certain cues are perceived, the risk of injury tends to be assessed in the form of a risk ratio: for example, the player then appears to be twice as at risk as usual or significantly more susceptible to injury than others in the training group. Such assessments are based on a combination of intuition, experience and situational contextual perception and therefore form an important basis for practical risk prevention in competitive sport.

### Clinical implications

The interviewed experts did not rely on absolute probabilities, but rather on relative risk judgments informed by their previous experiences and situational impressions. Such assessments are often based on observable cues that gain meaning through their interpretation within the broader higher-order themes emerging from stakeholders' experiences. These heuristics, while valuable, are difficult to formalise or automate, particularly when dealing with dynamic and psychological factors such as pressure, fatigue, or motivation.

In practical sports settings like competitive badminton, where sample sizes are small and individual variability is high, injury prevention should therefore not depend solely on rigid, standardised thresholds derived from data. Instead, a hybrid approach is recommended [49]: combining subjective human insight with interpretable, flexible data models that can be adjusted and enriched through expert input. Interpretable machine learning systems, for instance, could allow experts to integrate contextual information into data-driven frameworks without losing transparency. Rather than replacing human judgment, such models should aim to support and extend it. To better understand the criteria of the coaches’ eye and to be able to transfer them into a transparent risk assessment, it would therefore be particularly important for stakeholder from the practice to try to specify and communicate their criteria. This will help to bridge the gap between informal expertise and formal modelling and provide a more holistic and practical tool for risk assessment and injury prevention in high performance environments.

Building on this perspective, one potential avenue for future development could involve the structured documentation of qualitative cues within existing athlete monitoring systems. For example, periodic athlete self-reports or structured coach observations could be logged alongside quantitative load and epidemiological data. Such entries might include perceived fatigue patterns, communication shifts or contextual stressors identified in this study.

If systematically recorded, these qualitative indicators could serve as complementary ‘flags’ that prompt closer interdisciplinary review when combined with quantitative risk markers. However, it is important to emphasize that the present study does not provide empirical evidence that such integration would improve injury risk prediction. Whether the structured incorporation of qualitative insights meaningfully enhances early detection of injury vulnerability remains to be prospectively verified.

### Limitations

Although the requirements for a successful implementation of a risk assessment model are different (insight into the causal structure that determines the risk of injury, predictive accuracy, evidence for the efficacy and effectiveness of preventive measures), they face the same central methodological challenge: the limited number of cases in high-performance sport. To attenuate this situation, this study incorporates the empirical knowledge and human judgement of various badminton experts in practice. However, a limited number of interviewees can limit the reproducibility of the higher-order themes and cues identified. The limitation in this study was due to the strict inclusion criteria (minimum playing level and duration of professional experience at this level) and was preferred over a higher number of participants. In addition, the level of agreement with the identified themes was high, reducing the risk of spurious results. This high level of agreement is also the reason why we did not initiate any further interviews after the 20 planned interviews. Braun and Clarke [31] do not strictly specify how many interviews should be included, but suggest a minimum number of six interviews and 15 interviews for scientific publications. This was achieved with a total of 20 interviewees. In addition, we made sure that an ‘occupational group’ was not only interviewed once, but that at least two views and experiences were described.

The inclusion of stakeholders in this study was based on the criterion of experiential knowledge specifically related to the transition of young athletes to elite athletes within the national badminton performance system. Although various stakeholders, such as psychologists and nutritionists, generally play an important role in supporting athletes, their involvement in the performance structures accessible for this study was limited. Consequently, they were less directly involved in the longitudinal transition processes and were therefore not systematically included.

There are only a few women who hold coaching or supervisory positions in German badminton. For this reason, we were only able to include a small number of women in the study. The limited representation of female participants and certain interest group roles may limit the transferability of the results, especially given potential gender differences in injury patterns and development trajectories.

When interpreting our results, it should be noted that the relationship between injuries and risk constellations was analysed in a high-performance environment. Firstly, this means that we are dealing with a highly selected and trained group of players in terms of both performance level and past injuries (which would lead to early retirement from competitive sport). Secondly, this means highly qualified and experienced medical and badminton-specific personnel. This limits the variability, for example, in the training history (as otherwise this level of performance could not have been achieved) or in the athletic condition of the players.

One final limitation is that, although we included all relevant experts, we did not include our exact target group (16–20-year-olds). Only one of the athletes we interview is within this target age group. The sampling strategy was intentionally designed to capture experience-based knowledge, that is, reflective insights derived from completed or repeatedly observed transition processes. We therefore prioritized individuals who had already navigated this developmental phase or had observed it across multiple athletes over time. Nevertheless, retrospective reports may be subject to recall bias. Furthermore, athletes currently undergoing the junior-to-elite transition may provide valuable in-the-moment perspectives that differ from retrospective reflections. Future research should therefore complement the present findings by investigating the lived experiences of athletes who are presently in this transitional phase.

## Conclusion

A considerable number of different higher-order themes and cues for an increased risk of injury at the transition age from junior to elite level were identified. These include signs of over-motivation resulting from high internal and external pressure to succeed and time pressure, an unprofessional attitude, which primarily results in a deficient athletic condition, as well as a lack of continuity in the training process and major changes in both the private and sporting context. According to the experts, some of the criteria that indicate an increased risk of injury are latent and cannot be quantified directly. This mainly concerns deviations from an athlete's normal state or behaviour, both on a physical level (movement quality) and on a human/social behavioural level. To date at least, these factors cannot be captured by artificial intelligence with the same quality as by humans themselves, as they are based on intuition and the subjective experience of coaches and have the potential to create a more holistic profile of the athlete. The complexity and dynamic interplay of multiple risk factors in competitive sports suggests that effective injury prevention requires multidimensional, adaptive strategies based on both scientific evidence and expert intuition. Ongoing methodological refinement with close collaboration between researchers and practitioners should continue to be implemented.

## Supplementary Information

Supplementary Material 1.

## Data Availability

Data in anonymised form available on request from the authors.
