# Supplementary material for: From young aces to injury cases: a qualitative study of stakeholder perspectives on injury risk in junior to elite badminton
Source: Sports Med Open. 2026 Oct 11;12:151. doi: 10.1186/s40798-026-01107-9 (PMC13653887; doi:10.1186/s40798-026-01107-9)
Supplement: Supplementary file 1 [file 40798_2026_1107_MOESM1_ESM.docx]

**From Young Aces to Injury Cases: A Qualitative Study of Stakeholder Perspectives on Injury Risk in Junior to Elite Badminton**

Brid Stepper^1,2^, Lars Donath^2^, Hendrik Stagge^2^, Oliver Faude^3^, Anne Hecksteden^1,4^

^1^ Institute of Sport Science, University Innsbruck, Innsbruck, Austria

^2^ Department of Training Intervention Research, German Sport University Cologne, Cologne, Germany

^3^ Department of Sport, Exercise and Health, University of Basel, Basel, Switzerland

^4^ Institute of Physiology, Medical University Innsbruck, Innsbruck, Austria

Correspondence to Anne Hecksteden: anne.hecksteden@uibk.ac.at

Journal name: Sports Medicine Open

# **Supplemental Material**

**Appendix A.** Interview protocol.

**Assessment of the injury risk of young athletes in the (high) competitive sport of badminton**

**Interview protocol**

**Name: _________________________­­­­­______________	Date: __________________**

1. **Declaration of consent**
2. **Introductory specifications**

- Reference group: young athletes in the sensitive transitional area (U17-U22) of German elite badminton
- Idea: Hypothetical ‘prediction tournament’: "Who can most accurately estimate the injury risk of the training group over the next 28 days? Who can predict who will get injured? What cues would you use?"
- Injury definition “index injury”: As soon as a unit cannot be performed due to physical complaints (= “time-loss”), this is considered an injury (both acute and overuse injuries)

3. **Initial personal assessments**

Comprehensive assessment of the risk of injury in badminton and the assessability of the individual injury risk of individual players.

In your own experience:

- How pronounced is the risk of injury in the transition area (U17-U22) in competitive badminton?
  - In comparison to other age groups?
  - In comparison to other sports?
- Is it possible to estimate the individual injury risk of a player (much more accurately than group-based indicators from the literature)?

4. **Free indication of significant cues**

Blanco cards

In your personal opinion and experience, what is the critical information for assessing the injury risk of an individual player at the transition age from junior to elite badminton?

5. **Selection from the prepared cue cards**

16 cards with ‘risk factors’ from the literature

6. **Free text specifications on the selected cards**

More detailed information on the selected/ named risk factors. Depending on the course of the conversation/ questions before or after placement.

7. **More detailed information on the selected/ named risk factors. Depending on the course of the interview/ queries before or after placement.**

Please arrange the cards on the table according to their importance. The vertical axis is used like a visual analogue scale.

8. **General framework**

Are there characteristics of a specific case that would greatly change the relative importance of the cues/arrangement of the cards? E.g. past injury, different culture, etc.

9. **Anecdotes**

Do you remember individual cases that have shaped your experience of the risk of injury in badminton? Would you share these “anecdotes” with us - without using names?

10. **Current “de facto” process at the training centre**

What is the current actual process here on this topic? Are you dealing with the question of whether there is a particular risk of injury to individual players or is this not actually so active?

11. **Potential for improvement**

Where do you see room for improvement? How could the risk be minimised?

12. **What kind of support for injury prevention and regeneration management would be helpful?**

What kind of digital support would be helpful to make better and safer decisions in injury prediction and thus injury prevention (e.g. rather an evaluation of individual risk factors or an integrative risk assessment)?

13. **Personal background**

- Training/ qualification as a coach
- Path into badminton/ own career
- Role of the coach in the rehab process
- How long have you been in this role?
- Career path
- Are you also scientifically active in badminton?

14. **Have we forgotten something important? Do you have any additional comments?**
15. **Thank you very much for your time and support!**

**Appendix B.** Terms on the cue cards (one term per card).

- Fitness and athleticism (prior to the current injury)
- Injury history
- Current injury / diagnosis
- Player’s age
- Gender
- Competition or training
- Personal playing style
- Discipline (singles, doubles, mixed doubles)
- Level of physical exhaustion
- Comorbidities (health issues unrelated to the current injury)
- Extrinsic factors (surface conditions, footwear, etc.)
- Dietary habits
- Level of mental fatigue
- External stress factors (school, personal life, etc.)
- Anatomy

**Appendix C.** Table of higher-order themes supplemented with direct quotations.

**Higher-order theme 1: Excessive pressure**

a) Sub-theme 1.1: Intrinsic pressure to perform

- ‘Above all, you want to prove yourself in the senior ranks as quickly as possible. I'm here, you can count on me. And when you're slowed down by an injury, you definitely feel more pressure.’

b) Sub-theme 1.2: Make it or not - external pressure in the transition age

- ‘Then there is certainly external pressure too, along the lines of ‘I want to play in the European Championships or World Cup this year and can't afford [an injury] right now, or I don't want to take a break because it might make a bad impression.’
- ‘(…) you didn't want to come across as weak and jeopardise a possible nomination, you didn't want to be someone who, for example, is injured every other week (…) and then you'd rather say, no, no, I'm fit, I'm going to see it through.'

**Higher-order theme 2: Recovery deficits**

a) Sub-theme 2.1: Accumulating load

- ‘(…) what I would say is that all these young people are training too hard at too early an age, and at some point their bodies will take revenge, especially when they reach the elite level. It works fine for a while, but at some point your body says, I've warned you five times, the sixth or seventh time will be the last. And then you have your first injury.’
- ‘Then the problem is often that when you move from the junior to the elite level, you have to play a lot of tournaments to prove yourself. And if you get injured at that point, you don't stand a chance, because (...) I think you have two or three years to establish yourself in the elite senior level.’

b) Sub-theme 2.2: Boom-and-Bust Cycle

- '(...) Then they say, “Yeah, it hurts a bit, but when it’s warm, it’s fine.” You know, the usual story. Then they either take a break for a while or do some light training, but they make the mistake of getting back into it far too quickly and end up reaching the point where they can’t do it at all.'

**Higher-order theme 3: Unprofessional attitude**

a) Sub-theme 3.1: Enthusiasm for athletics training is key

- ‘If you are good at both [mobility and strength], then you can slip and still recover well. If you are not, then you will fall and get injured.’
- ‘Well, I believe that athletic training is essential. I think that's a fact, that it has to be done. And of course, the more resources you have, the more individually you can work. I think that's important in the end, because athlete A is not the same as athlete B, and if everyone does squats with 120 kilograms, that's okay for one person but not for another.’

b) Sub-theme 3.2: Lacking athletic mindset

- ‘But she’s just not an athlete with the right mindset, and maybe she doesn’t even want to be one – she never has been – and you can tell her body isn’t built for it. And it really doesn’t matter whether she trains hard or takes it easy; then she plays in the league at the weekend (…) and bang, she gets injured, twists her ankle.’

**Higher-order theme 4: Challenges to the continuity of the training process**

a) Sub-theme 4.1: Life events

- ‘Moving from school to university. (…). So your environment changes. You have different external influences. You meet different people. And when you change your training centre after your youth, the risk of injury is much higher due to additional changes in your daily training routine.’
- ‘Yes, well, I would say that when you change training centres (…), when you move to a new environment and you are still relatively young and perhaps don't have the standing to prove yourself and don't dare to speak up when you have an injury, because you are basically happy and grateful to have been accepted into this group.’

b) Sub-theme 4.2: Interruptions in the training process

- ‘So, the most dangerous thing is when players suffer from (...) some kind of perceived time pressure. For example, a tournament is in 14 days. And now there is (...) some kind of incident, let's say they have a cold (...). And now there's this perceived deadline. And now they want to be fit at all costs and think they have to go the extra mile. That's when my alarm bells start ringing. So, when players don't give themselves time to build up properly, but just try to do everything at the last minute (...). Normally, they would know, okay, start slowly and so on. But that's not possible now because of the tournament, so they just go full throttle right away.’

**Appendix D.** Table of cues supplemented with direct quotations.

**Cue-family 1: Signs of over-motivation**

a) Cue 1.1: Players do additional training (“Trying with the crowbar”)

- ‘You want to show that you are there. You want to show that you are ready to go. You want to show that, ideally, you can do more than the others.’

b) Cue 1.2: Lack of honest communication

- ‘Because the question is always how high your self-motivation is, not to mention. If you're 17 and mentally and physically exhausted, but you want to make it onto the national team, you're not going to say, ‘Hey, coach, I need a day off, I can't do anything today, I'm exhausted.’ You (...) suggest that you can still keep going.’
- ‘(…) that they don't always tell me everything. Of course, they want to train. I understand that; they probably want to show the coach what they can do. I understand all that. But it doesn't help if we train to the point where we can't go any further.’

**Cue-family 2: Unprofessional attitude (‘not being an athlete’)**

a) Cue 2.1: Visibly deficient athleticism

- ‘So, I think, you look at the basic constitution of an athlete. Does he look very athletic? You just look, is the muscular well defined? Or is it someone who (...) is rather flabby, soft (...). Where you think, you would like to be a bit more defined. But I don't know if there are any studies that would really show that. It's just more of a subjective gut feeling.’

b) Cue 2.2: Unhealthy lifestyle

- '(...) from what I’ve noticed with them, it’s really all about the quantity of sleep. As for sleep quality, they don’t have that many worries yet – they don’t have to process anything – they just go to bed and sleep. But before they do that, they usually spend a lot of time gaming and so on. And the next morning in training, I can tell straight away if they’ve had another short night.’

**Cue-family 3: “Something is different”**

a) Cue 3.1: Visible (rapid) changes to the body

- ‘And that was actually a very exciting phase, because you could have seen it. At that time, I had done a nutritional consultation beforehand and had strictly adhered to this diet plan for six weeks and really lost weight. And then (...) I was training in the morning and I twisted my ankle.’

b) Cue 3.2: Deviation from habitual movement quality

- ‘What features would I look out for? How they move. Based on their own movement profile. You know that after years. I can't describe it very well. If I see that someone isn't moving well, then I would recognise that relatively well, I would say.’
- ‘(...) when they're on the court (...) I think you can see that quite well. Then you think, what's wrong with him?’

c) Cue 3.3: Psycho-social deviations from the ‘individual normal state’

- ‘So with me, I always try to greet the players personally in the morning. Look at them, say good morning. And when I notice something about the players' appearance or behaviour - sometimes you can tell by the time they come to training - that sets alarm bells ringing.’
- ‘Movement quality, mindset, motivation, how clear their gaze is, how annoyed they get when they make mistakes. If he's romping around today (...), then I think, yes, has it [always] been like that? Then you know, okay, something's just not right. Unusual for him. That's what I mean by getting to know players, behaviour patterns. If a racket doesn't fly when it usually does, then he's either grown up or he's not 100% there. He more or less doesn't care (...).’

**Cue-family 4: Upheavals in the private life**

- ‘(…) sometimes you can tell by the time they come to training - that sets alarm bells ringing. If they arrive far too early, then I know it's a tournament, they're over-motivated. You have to observe them a bit. If they arrive at the last minute, then they've probably had a bad night's sleep (...) or something.’

**Cue-family 5: Exposure**

a) Cue 5.1: New stuff - The danger lies in the unknown

- ‘So if you want to do something new in training (...). Something where the players have to get out of the box. Then they suddenly get injured.’

b) Cue 5.2: Load spikes

- '(...) I don’t think I would have got through my career so injury-free without that athletic training. On the other hand, I’d say it had a massive impact on the start of my career – that is, the start of my Under-19 career – and initially a negative one at that. (…) I was able to put on a lot of weight, and my body could probably handle it, but then, combined with this and that, it was too much.'

**Cue-family 6: Signs of wear**

- ‘You always have a pinch somewhere, you always have something somewhere, I don't know if it's just before a strain or somehow a muscle hurts (...). But in 80% of cases, it's just that you have something, and that might be gone after a day or two. Whether it's a good thing that it's gone away through training is another matter.’
- ‘And then you have minor overloads, which then just add up. The problem is that such things are usually not treated by the coaches with the same care as if someone really has an ACL. (...). But such little things, where you say, well come on, join in again or something. This is, of course, something that adds up in the long run and then every now and then (...) causes failures.’

The unclear nature of many of the injuries sustained:

- ‘And then there are those where you thought, okay, that can't possibly go well, and then they're not injured at all afterwards. It's quite strange.’
- ‘So there are a lot of examples where it looks to me that the person will definitely get injured, and in others where everything actually looks totally smooth and round, I feel that no fewer injuries occur.’
- ‘In retrospect, you can always find something, but the bad luck component (...) is always there. That's just part of the risk of doing sports. And in competitive sports, we work with fatigue and adaptation and try to push people to their limits. Let me put it this way: if you play badminton every day, the likelihood of you getting injured is higher than if you play badminton once a month. That's simply the point.’

**Appendix E.** Frequency table of higher-order themes across stakeholder interviews.

Frequencies indicate the number of interviews in which a theme was identified and are presented to increase transparency regarding the distribution of themes across the dataset. Although each interviewee named or expressed the respective theme slightly differently, it could be unambiguously related to the corresponding theme. Several sub-themes could be mentioned by a single interview partner.

| **Theme/ sub-theme** | **Frequency of mention (number of interviews)** | **Percent** |
| --- | --- | --- |
| Higher-order theme 1: Excessive pressure | Σ 22 |  |
| Sub-theme 1.1. Intrinsic pressure to perform | 14 | 70% |
| Sub-theme 1.2. External pressure | 8 | 40% |
| Higher-order theme 2: Recovery deficits | Σ 19 |  |
| Sub-theme 2.1. Accumulating load | 13 | 65% |
| Sub-theme 2.2. Boom-and-Bust Cycle | 6 | 30% |
| Higher-order theme 3: Unprofessional attitude | Σ 21 |  |
| Sub-theme 3.1. Enthusiasm for athletics training is key | 13 | 65% |
| Sub-theme 3.2. Lacking athletic mindset | 8 | 40% |
| Higher-order theme 4: Challenges to the continuity of the training process | Σ 13 |  |
| Sub-theme 4.1. Life events | 8 | 40% |
| Sub-theme 4.2. Interruptions in the training process | 5 | 25% |
| Total number of interviews analysed | 20 | 100% |

**Appendix F.** Frequency table of cue families across stakeholder interviews.

Frequencies indicate the number of interviews in which each cue family was identified and are provided for transparency only. Although each interviewee named or expressed the respective cue slightly differently, it could be unambiguously related to the corresponding theme. Several cues could be mentioned by a single interview partner.

| **Cue-family/ cue** | **Frequency of mention (number of interviews)** | **Percent** |
| --- | --- | --- |
| Cue-family 1: Signs of over-motivation | *Σ 20* |  |
| Cue 1.1: Players do additional training | 13 | 65% |
| Cue 1.2: Lack of honest communication | 7 | 35% |
| Cue-family 2: Unprofessional attitude | *Σ 19* |  |
| Cue 2.1: Visibly deficient athleticism | 13 | 65% |
| Cue 2.2: Unhealthy lifestyle | 6 | 30% |
| Cue-family 3: “Something is different” | *Σ 26* |  |
| Cue 3.1: Visible (rapid) changes to the body | 5 | 25% |
| Cue 3.2: Deviation from habitual movement quality | 12 | 60% |
| Cue 3.3: Psycho-social deviations from the ‘individual normal state’ | 9 | 45% |
| Cue-family 4: Upheavals in the private life | *13* | 65% |
| Cue-family 5: Exposure | *Σ* 21 |  |
| Cue 5.1: New stuff - The danger lies in the unknown | 11 | 55% |
| Cue 5.2: Load spikes | 10 | 50% |
| Cue-family 6: Signs of wear | 10 | 50% |
| Total number of interviews analysed | 20 | 100% |
